# Testing of library preparation methods for transcriptome sequencing of real life glioblastoma and brain tissue specimens: A comparative study with special focus on long non-coding RNAs

**DOI:** 10.1371/journal.pone.0211978

**Published:** 2019-02-11

**Authors:** Marek Vecera, Jiri Sana, Jan Oppelt, Boris Tichy, Kopkova Alena, Radim Lipina, Martin Smrcka, Radim Jancalek, Marketa Hermanova, Leos Kren, Ondrej Slaby

**Affiliations:** 1 Central European Institute of Technology (CEITEC), Masaryk University, Brno, Czech Republic; 2 Department of Neurosurgery, University Hospital Ostrava, Ostrava, Czech Republic; 3 Department of Neurosurgery, University Hospital Brno, Faculty of Medicine, Masaryk University, Brno, Czech Republic; 4 Department of Neurosurgery, St. Anne’s University Hospital and Faculty of Medicine, Masaryk University, Brno, Czech Republic; 5 1st Department of Pathological Anatomy, St. Anne’s University Hospital and Faculty of Medicine, Masaryk University, Brno, Czech Republic; 6 Department of Pathology, University Hospital Brno, Faculty of Medicine, Masaryk University, Brno, Czech Republic; 7 Department of Comprehensive Cancer Care, Masaryk Memorial Cancer Institute, Faculty of Medicine, Masaryk University, Brno, Czech Republic; Baylor University Medical Center, UNITED STATES

## Abstract

Current progress in the field of next-generation transcriptome sequencing have contributed significantly to the study of various malignancies including glioblastoma multiforme (GBM). Differential sequencing of transcriptomes of patients and non-tumor controls has a potential to reveal novel transcripts with significant role in GBM. One such candidate group of molecules are long non-coding RNAs (lncRNAs) which have been proved to be involved in processes such as carcinogenesis, epigenetic modifications and resistance to various therapeutic approaches. To maximize the value of transcriptome sequencing, a proper protocol for library preparation from tissue-derived RNA needs to be found which would produce high quality transcriptome sequencing data and increase the number of detected lncRNAs. It is important to mention that success of library preparation is determined by the quality of input RNA, which is in case of real-life tissue specimens very often altered in comparison to high quality RNA commonly used by manufacturers for development of library preparation chemistry. In the present study, we used GBM and non-tumor brain tissue specimens and compared three different commercial library preparation kits, namely NEXTflex Rapid Directional qRNA-Seq Kit (Bioo Scientific), SENSE Total RNA-Seq Library Prep Kit (Lexogen) and NEBNext Ultra II Directional RNA Library Prep Kit for Illumina (NEB). Libraries generated using SENSE kit were characterized by the most normal distribution of normalized average GC content, the least amount of over-represented sequences and the percentage of ribosomal RNA reads (0.3–1.5%) and highest numbers of uniquely mapped reads and reads aligning to coding regions. However, NEBNext kit performed better having relatively low duplication rates, even transcript coverage and the highest number of hits in Ensembl database for every biotype of our interest including lncRNAs. Our results indicate that out of three approaches the NEBNext library preparation kit was most suitable for the study of lncRNAs via transcriptome sequencing. This was further confirmed by highly consistent data reached in an independent validation on an expanded cohort.

## Introduction

The rise of next-generation RNA sequencing (NGS or RNASeq) of transcriptome has largely accelerated genomic and epigenetic research and allowed the discovery of new RNA species which could harness a potential to be useful in the study of particular diseases and the development of new therapeutic strategies [1, 2]. However, as is usually the case during implementation of new methods, there are issues that need to be tackled with in order to maximize the accuracy and the value of gathered experimental data. One such issue is the selection of a proper approach to molecular library preparation and sequencing with regard to the molecular targets of interest [3–5].

Our aim is to study transcripts called long non-coding RNAs (lncRNAs), which do not code for protein and, instead, perform as fully functional regulatory elements which have been implicated in many biological processes including transcriptional regulation of neighbouring or distant genes [6] and histone modification [7], and those related to malignancies such as gliomas [8, 9]. Many lncRNAs are antisense to coding genes, intergenic or intronic [10] and, thus, suitable library prepration protocol needs to reflect this fact and retain strand-specific information. This can be achieved via incorporation of deoxyuridine triphosphates (dUTPs) during the synthesis of the second strand which is later cleaved using uracil-DNA glycosylase [11, 12] or via starter/stopper binding sites determining the insert size and modulating the activity of reverse transcriptase [13]. Another important aspect which needs to be looked at is the target molecule recovery of NGS after comparing processed and mapped reads to an annotated database such as Ensembl [14]. The goal is to achieve the highest numbers of target molecules captured via NGS; both mRNAs and lncRNAs in the case of our current studies. Each method of library preparation may produce significantly different numbers based on the applied technology and steps of the procedure. This particular aspect could render some of the library preparation methods not suitable for the study of lncRNAs since they by default principle are somehow limited or biased towards other RNA species, for example genes from coding regions of the genome. Last but not least, other factors such as the sequencing depth, eveness of transcript coverage and the duplicate rates need to be also considered in order to find the most suitable approach which would produce sequencing data with sufficient sequencing depth, even transcript coverage from start to end [15] and low rates of artificial duplicates introduced during the construction and amplification of libraries [16]. Otherwise, sequencing of libraries prepared with improper approach would result in the permanent loss of valuable data about lowly expressed lncRNAs.

We used 3 library preparation kits to generate 9 libraries from glioblastoma (GBM) and non-tumor (NonT) samples sequenced in Core Facility Genomics (CFG) at CEITEC MU and compared their performance on the basis of abovementioned factors. The results generated with the best performing kit were then independently validated in an expanded cohort of patient samples in Vienna Biocenter (VB) Core Facility NGS Unit.

## Results

### RNA quantification and quality control

The concentration, purity and RNA integrity number (RIN) of total RNA samples are listed in Table 1. Pure RNA is characterized by A_260_/A_280_ ratio being approximately 2.00 which is the case of all samples indicating no protein contamination. The second ratio is expected to be 2.00–2.20 which was the case of all but three samples (GBM-1, NonT-1, NonT-3) whose low A_260_/A_230_ ratio signifies some degree of contamination with organic contaminants which absorb at 230 nm, for example acidic phenol. RIN of RNA samples varied between 5.5 and 9.2.

**Table 1:** Concentration, purity and RNA integrity number (RIN) of isolated and diluted total RNA samples. Purity of RNA was evaluated based on absorbance ratios which should be approximately 2.0. Lower A_260_/A_230_ hints at the presence of organic contaminants, for example acidic phenol. RNA with sufficient integrity for all downstream applications is characterized by RIN 8.0 or more.

RNA Sample	Concentration (Qubit) [ng/μl]	A_260_/A_280_	A_260_/A_230_	RIN
GBM-1	242.2	2.05	1.84	9.2
GBM-2	216.4	2.09	2.00	5.5
GBM-3	203.7	2.06	2.00	8.4
GBM-4	227.0	2.10	2.11	9.0
GBM-5	246.1	1.98	2.01	8.5
NonT-1	211.4	2.00	1.95	7.7
NonT-2	261.5	2.06	2.11	7.7
NonT-3	282.1	2.06	1.05	7.9

**Fig 1:**
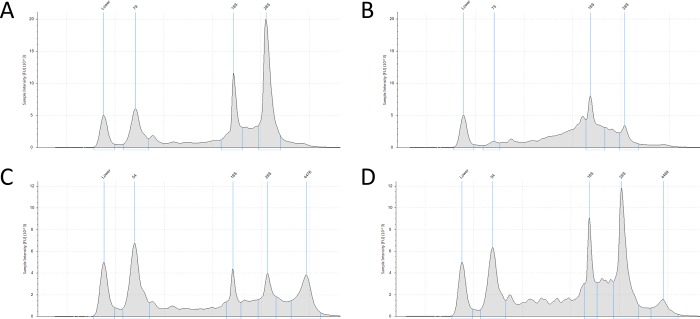
Four examples of electropherograms (EPGs) of total RNA samples analyzed on 2200 TapeStation. In this figure, x-axis represents the number of base pairs. The position of other peaks is derived from the position of the first peak (lower marker). Peaks 18S a 28S represent ribosomal (rRNA) subunits. Peak in the position of ~80 bp represents short RNA species and the last peak in the position of 15–16 kbp represents genomic DNA (gDNA). **A)** EPG of GBM-1 contains proportional peaks for 18S and 28S subunits indicating high integrity of the sample and no visible peak in the position of 15–16 kbp signifying no gDNA contamination. **B)** EPG of GBM-2 indicates low integrity of the RNA sample judging by the severe degradation of 28S and resulting disproportionality of height of both rRNA peaks. Again, gDNA was not observed. **C)** EPG of NonT-1 depicts 18S and 28S peaks with approximately same height which, again, is the mark of advanced degradation. Similar abundance of gDNA was also observed. **D)** EPG of NonT-3 shows fairly intact RNA with minor but visible gDNA contamination.

Examples of electropherograms (EPGs) of RNA samples with different level of integrity (18S/28S peak area ratio) or degree of genomic DNA (gDNA) contamination are depicted in Fig 1. Samples of sufficient quality were expected to have two visible peaks of proportional height representing 18S and 28S ribosomal (rRNA) subunits, a small peak in the position of ~80 bp representing short RNA species and no visibile peak in position of ~15–16 kbp which would indicate gDNA contamination. Such was the case of samples GBM-1, GBM-3 and GBM-4. All RNA samples contained both rRNA subunits in observable amounts with different proportionality denoted by RIN. In the case of sample GBM-2, the 28S rRNA subunit was significantly degraded compared to 18S which resulted in a considerably low RIN of 5.5. Three RNA samples (GBM-5, NonT-1, NonT-3) contained varying levels of gDNA which was later removed by DNase treatment.

### Descriptive parameters of molecular libraries

The concentrations, modal lengths and calculated molarities of 9 CFG libraries and 8 VB libraries are listed in Table 2. The concentrations of libraries were highly variable which was dependent on the input amount of rRNA-depleted RNA and the number of PCR cycles which was set according to the manufacturers' protocols. The modal lengths of each triad of libraries differed depending on the employed method of generating fragments from input rRNA-depleted RNA and its integrity. While the modal length of NEXTflex (NF) libraries was ~390 bp, the application of the SENSE kit (LX) and NEBNext kit (NB and VB) resulted in lower numbers.

**Table 2:** Concentration, modal length and molarity of molecular libraries. Nine CFG libraries were generated with three different library preparation kits NEXTflex (NF), SENSE (LX) and NEBNext (NB) and eight VB libraries were independently prepared with NEBNext kit. Concentration was measured on Qubit 2.0 and modal lengths were determined using 2200 TapeStation or Fragment Analyzer. Molarity was calculated from these two parameters using an online weight-to-moles conversion calculator [24].

**Library**	**NF**			**LX**			**NB**		
	**Conc.**	**Modal length**	**Molarity**	**Conc.**	**Modal length**	**Molarity**	**Conc.**	**Modal length**	**Molarity**
	ng/μl	bp	nM	ng/μl	bp	nM	ng/μl	bp	nM
G1	1.70	395	6.63	1.77	309	8.83	6.11	291	30.67
G2	2.02	397	7.84	0.56	331	2.61	10.60	275	59.39
N1	3.49	390	13.79	0.31	280	1.71	5.80	342	26.13
**Library**	**VB**								
	**Conc.**			**Modal length**			**Molarity**		
	ng/μl			bp			nM		
G1_VB	7.4			305			37.38		
G2_VB	15.3			322			73.21		
G3_VB	6.9			302			35.20		
G4_VB	7.9			306			39.78		
G5_VB	3.4			303			17.29		
N1_VB	1.9			305			9.60		
N2_VB	10.0			308			50.03		
N3_VB	8.9			305			44.96		

### Quality control of raw sequencing reads and evaluation of GC content

The acquired NGS datasets were downsampled to 2.5 million reads, and they underwent quality control (QC) performed with FastQC. Percentage of duplicate reads ranged from 17.8% to 73.7% with the top three values belonging to all three libraries prepared from sample NonT_1. The average GC content in CFG libraries ranged from 47% to 54% with the top two values belonging to NB libraries G2_NB (53%) and N1_NB (54%). As for the VB libraries, the percentage of duplicate reads ranged from 25.0% to 51.6% and the average GC content was between 46% and 50% (Table 3).

**Table 3:** Percentage of duplicates and average GC content. Calculated for 9 CFG libraries prepared with 3 different library preparation kits NEXTflex (NF), SENSE (LX) and NEBNext (NB) and 8 VB libraries independently prepared with NEBNext kit.

**Library**	**NF**		**LX**		**NB**	
	**Duplicates**	**Average GC content**	**Duplicates**	**Average GC content**	**Duplicates**	**Average GC content**
	%	%	%	%	%	%
G1	24.0	49.0	30.5	49.0	20.9	49.0
G2	17.8	47.0	39.0	49.0	29.0	53.0
N1	46.6	50.0	73.7	48.0	60.6	54.0
**Library**	**VB**					
	**Duplicates**			**Average GC content**		
	%			%		
G1_VB	27.8			46.0		
G2_VB	25.0			46.0		
G3_VB	27.5			46.0		
G4_VB	29.0			46.0		
G5_VB	34.8			48.0		
N1_VB	51.6			50.0		
N2_VB	33.5			45.0		
N3_VB	36.9			47.0		

**Fig 2:**
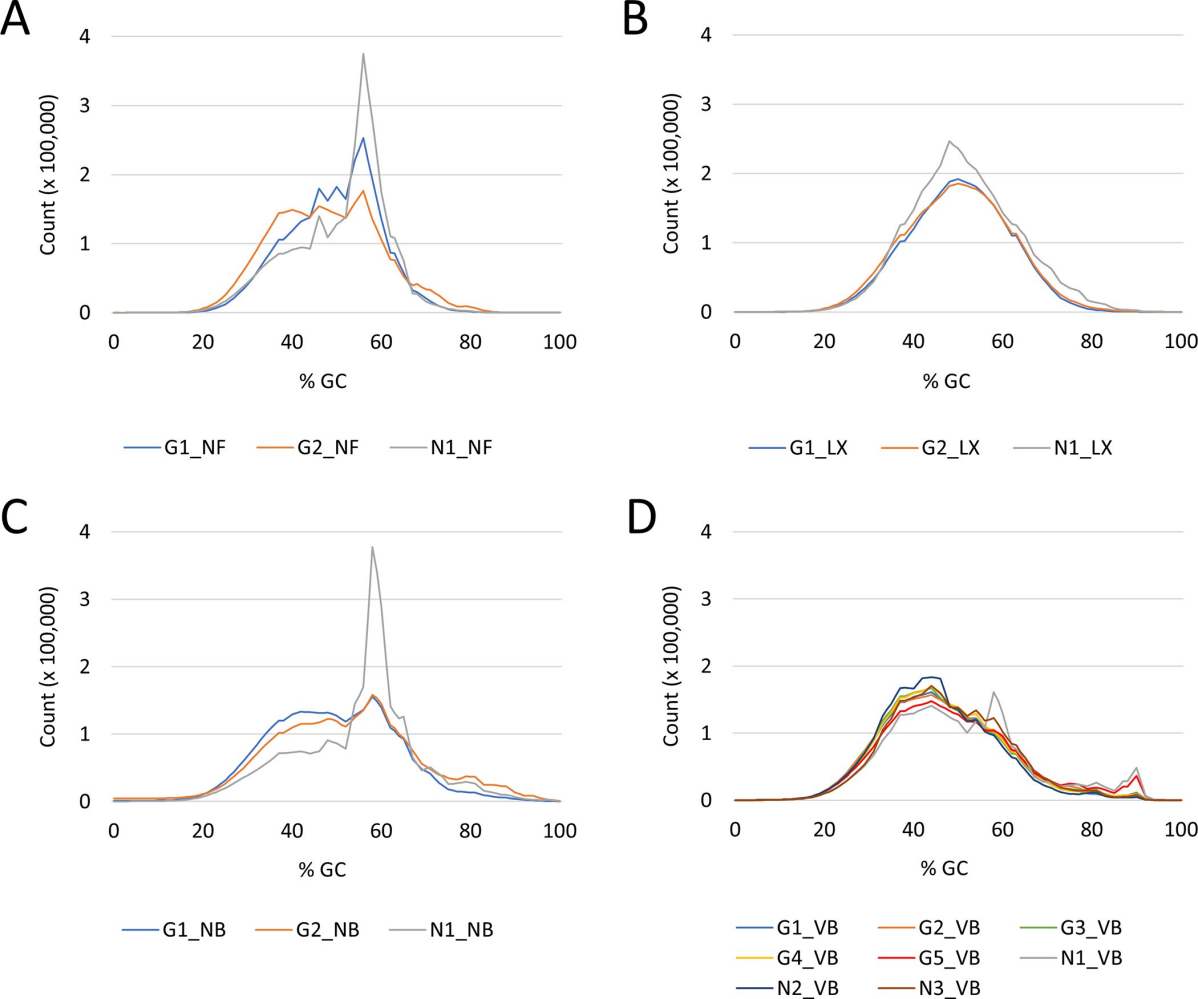
Normalized average GC content of reads. A roughly normal distribution of GC content is typical for normal random libraries. **A)** NEXTflex (NF) libraries do not have a normal distribution of GC content with sharp peaks present at the position of 55% indicating possible specific contamination, for example adapater dimers, or other bias. **B)** SENSE (LX) libraries exhibit normal distribution with the exception of library N1_LX based on the presence of a sharp peak around 50%. **C)** NEBNext (NB) libraries also do not have a normal distribution of GC content judging by the sharp peaks around 60% indicating the same problem as that of the NEXTflex libraries. **D)** NEBNext (VB) libraries have a roughly normal distribution of the GC content with the most distinct exception being N1_VB.

GC content was also measured across the whole length of each sequence in a given dataset, normalized per sequence (GC count per read) and plotted. A roughly normal distribution of GC content is typical for normal random libraries. Libraries most closely resembling the normal distribution were LX libraries G1_LX and G2_LX (Fig 2).

**Fig 3:**
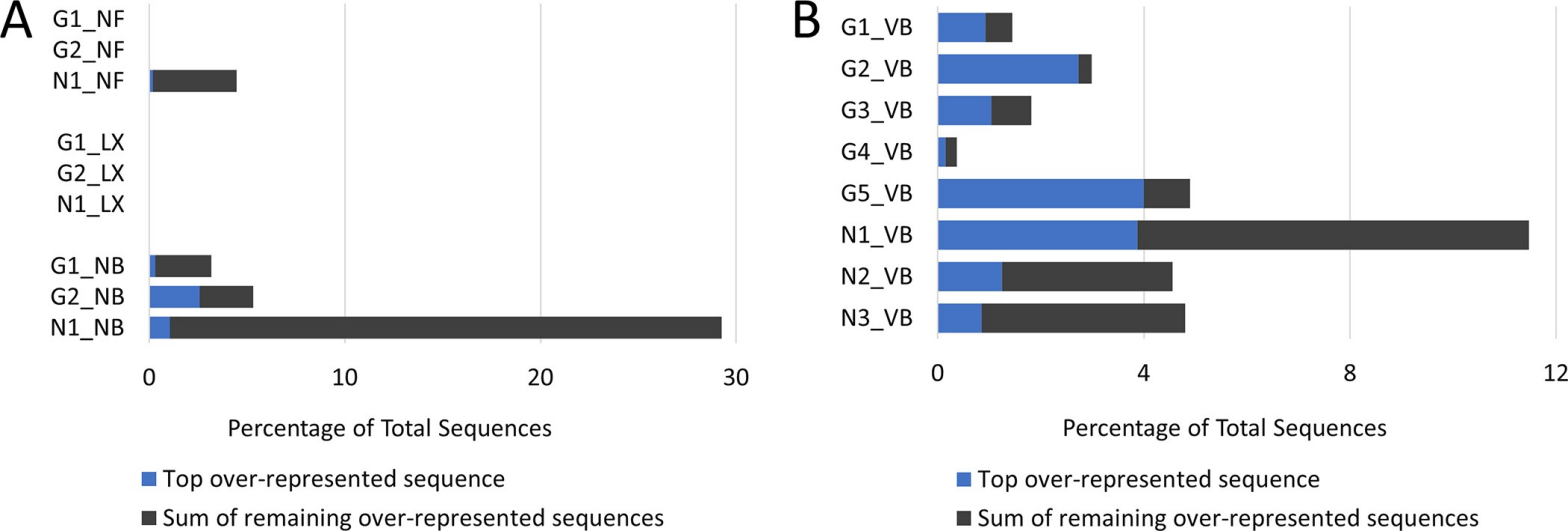
The total amount of over-represented sequences. The top frequent sequence is in blue. A) The numbers in CFG libraries vary with no over-represented sequences found in two NEXTflex (NF) libraries and all three SENSE (LX) libraries. The highest percentage of these sequences are present in N1_NB. **B)** VB libraries all contain over-represented sequences to some extent with the highest number belonging to N1_VB.

Adapter content in all libraries determined via FastQC was significantly low (≤ 0.1%). The amount of over-represented sequences was evaluated in every library again with FastQC which may indicate problems with ribosomal depletion or other bias. Out of CFG libraries, the highest presence of these sequences was found in N1_NB (29.25%), followed by G2_NB (5.32%), N1_NF (4.47%) and G1_NB (3.18%). No over-represented sequences were present in the rest of the libraries (Fig 3A). The highest amount of over-represented sequences was found in N1_VB (11.48%) which corresponds to CFG library N1_NB. Other VB libraries contained less than 5% of over-represented sequences. Interestingly, G1_VB and G5_VB contained predominantly one over-represented sequence (Fig 3B).

### Alignment and mapping statistics

**Fig 4:**
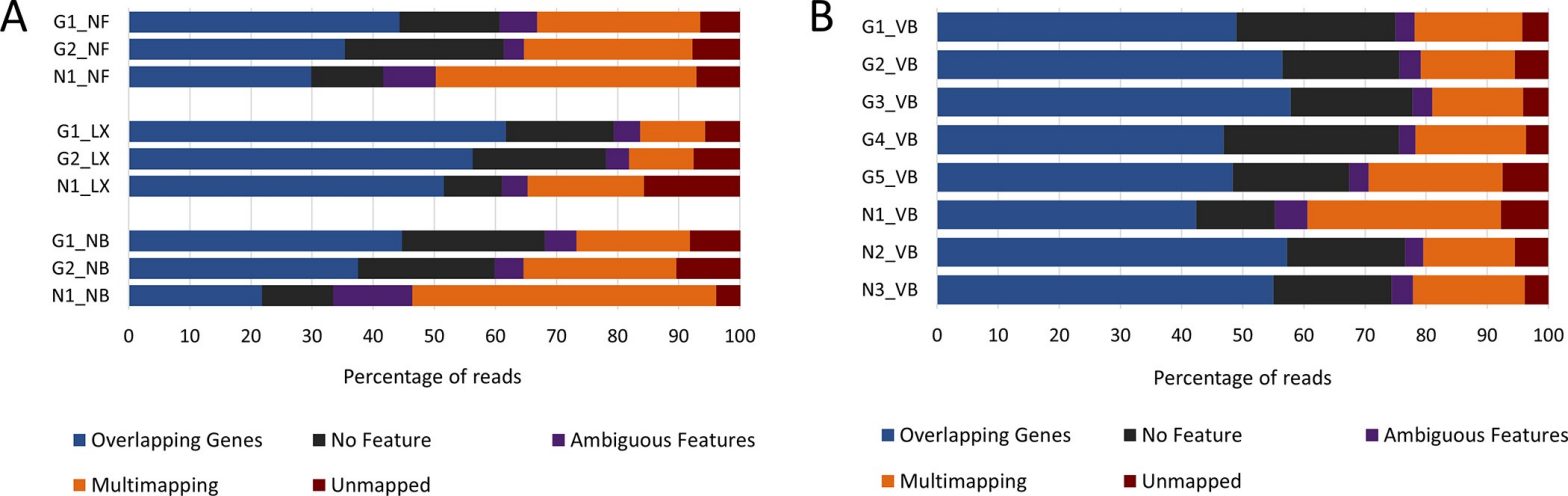
Statistics generated using Gene Counts mode of alignment tool STAR. The category Overlapping Genes includes sequences which overlap only one gene and together with the categories No Feature and Ambiguous Features comprise all uniquely mapped reads. Reads that were put into the category Multimapping do not map uniquely but to multiple loci, and the rest of the sequences could not be mapped at all, hence the category Unmapped. **A)** The highest numbers of reads in the category Overlapping Genes belong to all three SENSE (LX) libraries which also contain least reads that map to multiple loci. On the other hand, libraries generated with NEBNext (NB) and especially NEXTflex (NF) exhibit higher multimapping rates and lower numbers of reads overlapping only one gene. **B)** VB libraries generally show relatively consistent multimapping rates with the exception of N1_VB. The numbers of reads overlapping single genes range between 42.4% to 57.8%.

Reads from downsampled datasets were aligned to the reference genome using STAR [17] with the resulting numbers of overall uniquely mapped reads from CFG libraries ranging between 46.4% to 83.7% of all mapped reads with the top two highest values belonging to LX libraries. Moreover, between 21.8% and 61.7% of all reads were those that overlap one and only one gene (Fig 4A). Uniquely mapped reads from VB libraries comprised 60.6% and 81.0% of all mapped reads and the percentage of reads that overlapped only one gene ranged from 60.6% to 81.0% of all reads (Fig 4B).

**Fig 5:**
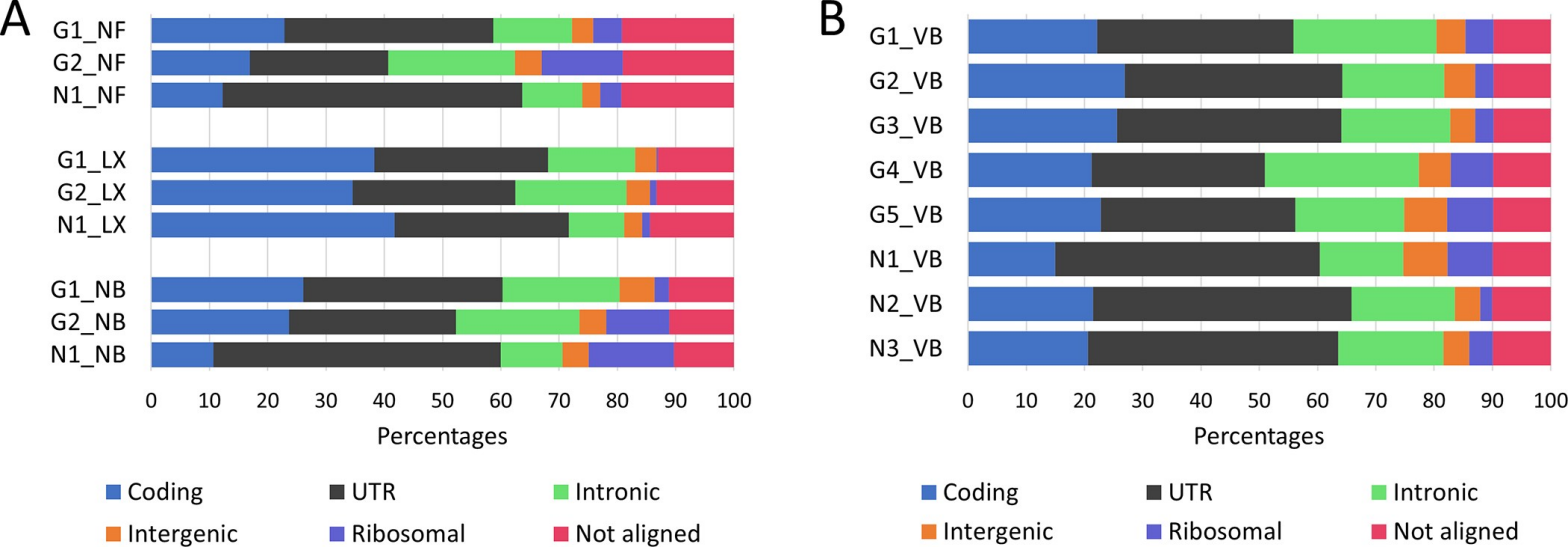
The percentage of reads aligned to particular regions in the reference genome. **A)** Among CFG libraries, SENSE (LX) libraries contained sequences which on average mapped more to coding regions and less to UTR compared to the other two kits. The number of bases aligning to intronic and intergenic regions ranged from 9.5 to 21.8% and 3.0 to 6.1%, respectively, with no major differences between the three groups of libraries. The highest numbers of bases aligning to ribosomal regions belonged to libraries G2_NB (11.1%), G2_NF (14.0%) and N1_NB (14.7%). **B)** VB libraries mapped to particular regions in a similar manner to NB libraries, as was expected, with the higher number of bases mapping to UTR compared to coding regions but with lower amount of base reads aligning to ribosomal regions.

The overall percentage of bases in primary alignments that align to particular regions in the reference genome calculated using Picard [18] was between 80 and 90% for CFG libraries and ~90% for VB libraries. While the SENSE kit seemed to prefer reads whose bases predominantly aligned to coding regions (>30%), the lowest numbers of bases in such primary alignments belonged to libraries N1_NF and N1_NB (10.7% and 12.2%, respectively). On the contrary, the numbers of base reads mapped to untranslated regions (UTR) were on average higher for NF and NB libraries. The number of bases aligning to intronic and intergenic regions ranged from 9.5 to 21.8% and 3.0 to 6.1%, respectively, with no major differences between the three groups of libraries. The top three highest numbers of bases aligning to ribosomal regions belonged to libraries G2_NB (11.1%), G2_NF (14.0%) and N1_NB (14.7%) (Fig 5A). The numbers of base reads from VB libraries mapped to coding regions ranged between 15.0% and 26.9% and those that were aligned to UTR regions ranged between 29.7% and 45.4%. The number of base reads mapped to intronic and intergenic regions ranged from 14.3 to 26.4% and from 4.3 to 7.6%, respectively. Finally, the number of bases mapped to ribosomal regions ranged from 2.0 to 7.9% (Fig 5B).

**Fig 6:**
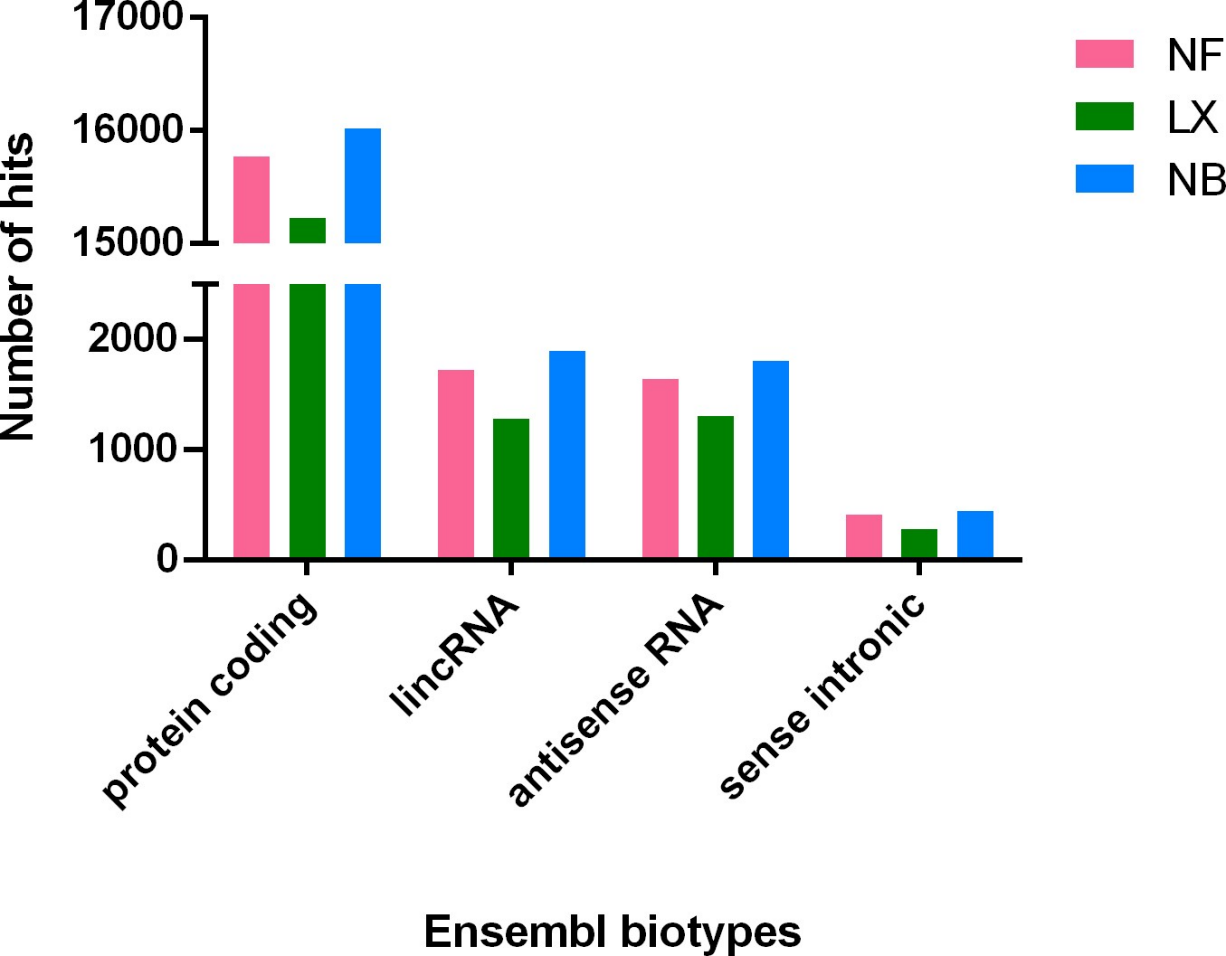
Results from comparison of three library preparation kits using the Ensembl automatic annotation system. The number of hits for each transcript biotype from Ensembl database calculated for each group of libraries generated using the same library preparation kit is on y-axis. Only the most populous biotypes relevant to our current study were included. While the first biotype comprises the majority of the group of protein coding RNA, the rest (lincRNA, antisense and sense intronic RNA) is part of another large group of long non-coding RNA. After comparing all three kits, the highest number of hits for each biotype of interest was observed for NEBNext (NB) libraries; NEXTflex (NF) libraries came second and SENSE (LX) libraries third. These results hint at a lesser variability of library fragments in LX libraries.

Uniquely mapped reads were also compared with Ensembl database using the Ensembl automatic annotation system [14, 19] and transcripts were classified into several biotypes which comprise larger groups. The most populous biotypes which are relevant to our current study are plotted in Fig 6, namely “protein coding”, “lincRNA”, “antisense RNA” and “sense intronic”. While the first is the major biotype of larger group of protein coding transcripts, the rest comprises group of lncRNA. Upon comparison of all three library preparation kits, we found that the highest number of hits for each biotype of interest belonged to NB libraries, while NF libraries came second and LX libraries third. This hinted at the lesser variability of library fragments generated with SENSE kit.

### Transcript coverage

**Fig 7:**
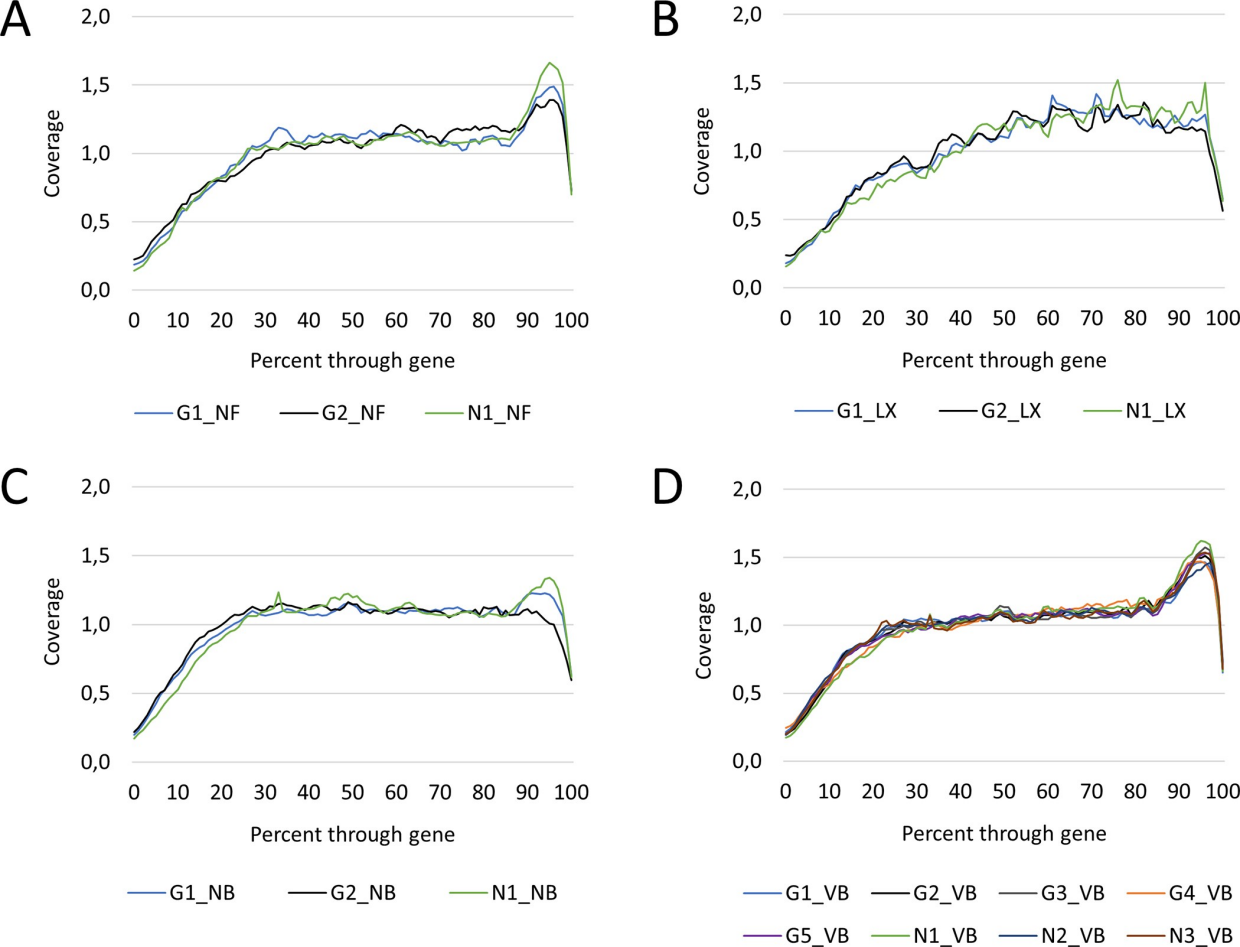
Normalized transcript coverage. **A)** NEXTflex (NF) libraries seemed to show relatively even transcript coverage with a notable peak in the 3' terminal parts of genes (~90–100%). **B)** Transcript coverage of SENSE (LX) libraries was more irregular hinting at a lesser variability of fragments generated by this particular kit. **C)** NEBNext (NB) libraries showed most even coverage with a notable peak in the coverage of 3' terminal parts of genes. **D)** NEBNext (VB) libraries showed even coverage similarly to NB libraries with more pronounced spike in the coverage of 3' terminal parts of genes.

Normalized gene coverage was calculated for all groups of libraries using Picard. Coverage of NF libraries seemed relatively even with notable peak in coverage of 3' terminal parts of genes (~90–100%) (Fig 7A). Coverage of LX libraries seemed more irregular hinting again at a lesser variability of fragments generated with this particular kit (Fig 7B). Relatively same even coverage was noted for NB and VB libraries again with the exception of the 3' terminal parts of genes where a spike in coverage could be observed in most of the libraries (Fig 7C and 7D).

### Efficiency of rRNA depletion and duplicate rates

Picard determined the percentage of rRNA ranging from as low as 0.3% to 17.9% with the three lowest values belonging to LX libraries G1_LX (0.3%), G2_LX (1.3%) and N1_LX (1.5%), and the highest value belonging to NF library G2_NF (17.9%). Significantly higher percentage of rRNA was also observed in NB libraries G2_NB (12.4%) and N1_NB (16.6%). The percentage of rRNA reads in VB libraries ranged between 2.2 and 8.8% and, thus, was even lower than in NB libraries. The duplicate rates for CFG libraries determined with Picard ranged from 25.6% to 91.5% with those belonging to LX libraries (55.1%, 59.3% and 91.5%) being significantly higher among each group of three libraries prepared from the same RNA sample. The duplicate rates for VB libraries ranged from 27.6% to 53.8% (Table 4).

**Table 4:** Percentage of ribosomal RNA (rRNA) and duplicate rates. Calculated via Picard tool for 9 CFG libraries generated with 3 different library preparation kits NEXTflex (NF), SENSE (LX) and NEBNext (NB) and 8 VB libraries independently prepared with NEBNext kit.

**Library**	**NF**		**LX**		**NB**	
	**rRNA**	**Duplicates**	**rRNA**	**Duplicates**	**rRNA**	**Duplicates**
	%	%	%	%	%	%
G1	6.1	43.1	0.3	55.1	2.8	25.6
G2	17.9	36.3	1.3	59.3	12.4	32.9
N1	4.6	63.2	1.5	91.5	16.6	65.1
**Library**	**VB**					
	**rRNA**			**Duplicates**		
	%			%		
G1_VB	3.5			29.6		
G2_VB	8.1			27.6		
G3_VB	5.3			29.9		
G4_VB	3.5			31.3		
G5_VB	8.8			35.4		
N1_VB	8.7			53.8		
N2_VB	2.2			35.0		
N3_VB	4.5			39.3		

**Fig 8:**
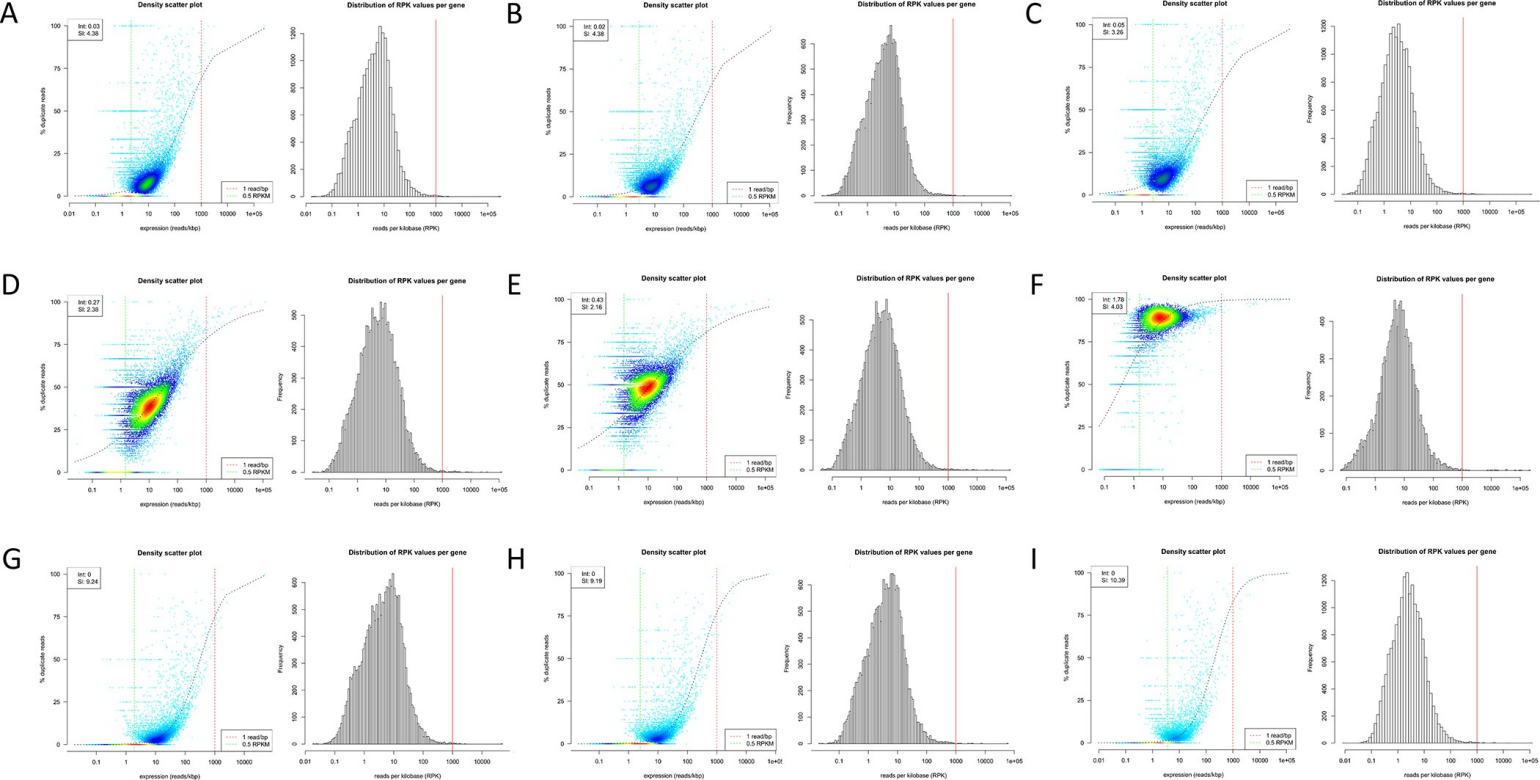
2D density scatter plots and the distribution of reads per kilobase (RPK) values per gene. Density scatter plots are relations of duplicate rates in % to expression in reads/kbp. Calculated using dupRadar for NEXTflex (NF) libraries **A)** G1_NF, **B)** G2_NF and **C)** N1_NF, SENSE (LX) libraries **D)** G1_LX, **E)** G2_LX and **F)** N1_LX, and NEBNext (NB) libraries **G)** G1_NB, **H)** G2_NB and **I)** N1_NB. While, duplication rates in NF and NB libraries were relatively low, LX libraries showed higher duplication rates. No skewed distributions of RPK values per gene with unusual amount of lowly expressed genes were observed.

**Fig 9:**
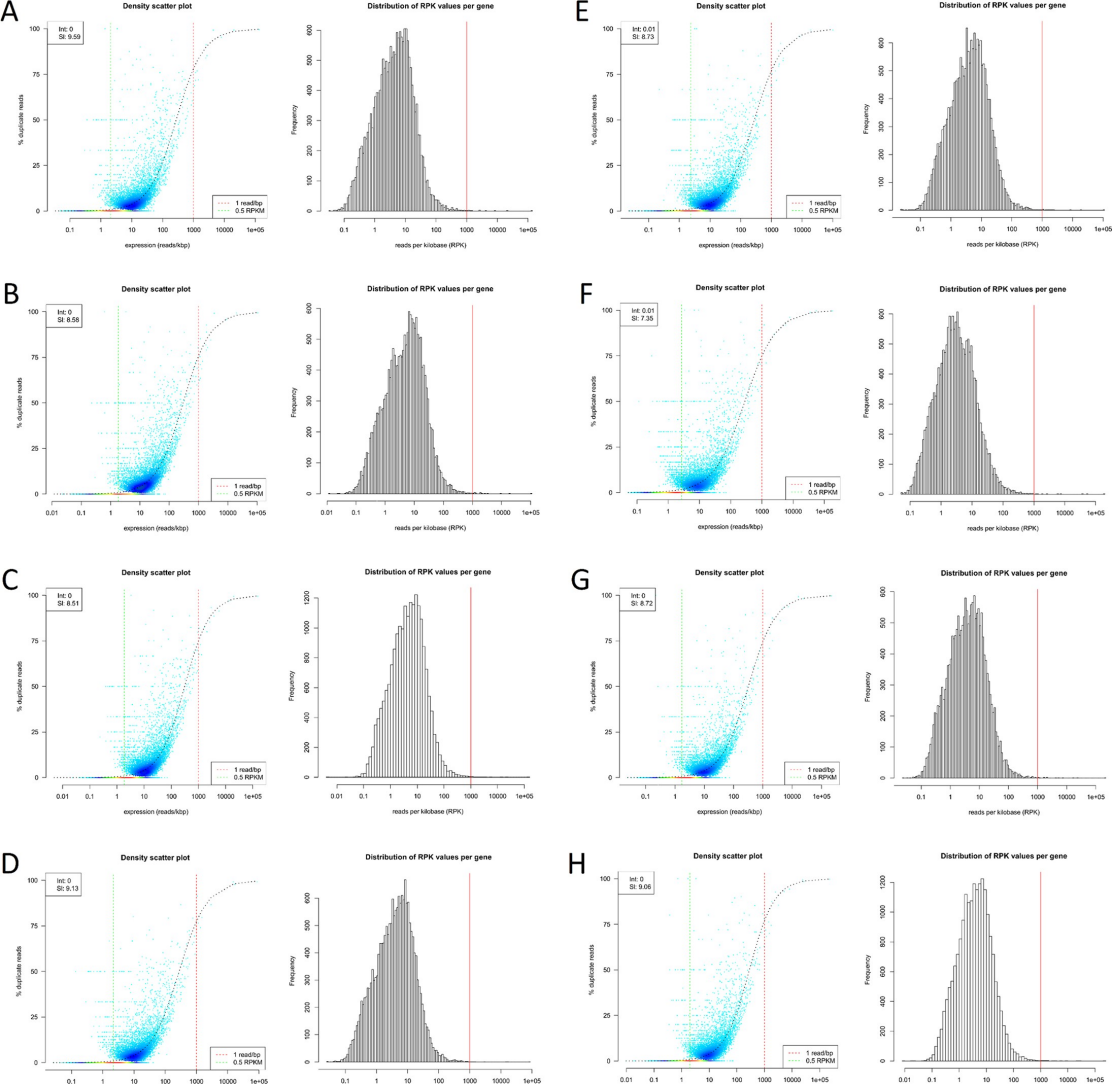
2D density scatter plots and the distribution of reads per kilobase (RPK) values per gene. Density scatter plots are relations of duplicate rates in % to expression in reads/kbp. Calculated using dupRadar for VB libraries **A)** G1_VB, **B)** G2_VB, **C)** G3_VB, **D)** G4_VB, **E)** G5_VB, **F)** N1_VB, **G)** N2_VB, **H)** N3_VB. Duplication rates in these libraries were relatively low and comparable to NB libraries prepared with the same library preparation kit NEBNext, as was expected. No apparent skewed distributions of RPK values per gene with unusual amount of lowly expressed genes were observed.

The 2D density scatter plots and histograms of the distribution of reads per kilobase (RPK) values per gene corresponding to each library generated using dupRadar [20] are depicted in Figs 8 and 9. Density scatter plots are relations of duplicate percentage to expression in reads/kbp. While NF and NB libraries showed relatively low duplicate rates, significantly higher numbers were observed in LX libraries, which was consistent with calculations from Picard. Moreover, duplication rates of NB libraries were comparable to those of VB libraries, as was expected. No apparent skewed distributions of RPK values per gene with unusual amount of lowly expressed genes were observed.

### Summary score

The most important results of comparative study are summarized in Table 5. The most suitable library preparation kit for NGS of lncRNAs reached the highest total scoring.

**Table 5:** Summary scores of chosen parameters of all three tested library preparation kits. Scores were assigned in four categories, specifically the percentage of rRNA reads and duplicate reads and the number of hits in Ensembl database for the groups of mRNAs and lncRNAs. The lowest score in each category is denoted by “-” and the highest by “++”. Based on these parameters, the most suitable kit for preparation of libraries for sequencing of lncRNAs, NEBNext, would be chosen and later independently tested in another NGS facility.

Parameter	NEXTflex (Bioo Sc.)	SENSE (Lexogen)	NEBNext (NEB)
% rRNA	-	++	+
% duplicates	+	-	++
mRNA (number of hits)	+	-	++
lncRNA (number of hits)	+	-	++
**Score**	**3+**	**2+**	**7+**

## Discussion

The fact that the prognosis of patients with GBM remains very poor to this day despite the available therapy poses a serious problem. Therefore, finding both new therapeutic targets and prognostic, predictive or diagnostic biomarkers is of utmost importance for making progress. One such candidate is the class of non-coding transcripts called lncRNAs which has been shown to regulate various biological processes related to GBM biology, including angiogenesis, epigenetic silencing, and alkyl agents and ionizing radiation resistance. In order to fully understand their value, deeper understanding of lncRNAs in GBM biology is vital. One of the approaches to finding potential novel biomarkers is the differential sequencing of transcriptomes of GBM patients and healthy controls which would reveal lncRNAs with significantly different expression between the two cohorts. This could indicate their importance in the biology of GBM where they could function as oncogenic transcripts or tumor suppresors. Thus, choosing the best approach for generating molecular libraries of sufficient quality suitable for the study of lncRNAs is fundamental.

The aim of our study was to compare three molecular library preparation kits NF, LX and NB for the sequencing of patient transcriptomes and evaluate their performance based on chosen parameters with the focus on lncRNAs. Three RNA samples with varying integrity have been chosen in order to see how well the library preparation methods would deal with this particular issue. The first stark difference observed before alignment of reads to the reference genome was the distribution of normalized average GC content which would reveal whether generated libraries were random or biased. This bias could indicate a specific contamination by over-represented sequences [21], most likely rRNA since the adapter content in all libraries was ≤ 0.1%. The most uneven distribution of GC content was observed in all three NF libraries whereas LX libraries showed roughly normal distribution. This would also later correlate with the percentage of rRNA reads which was generally high for NF libraries and the lowest for LX libraries. NB libraries showed relatively even distribution with the noticeable exception of N1_NB which again showed biased GC content distribution with unusually high normalized average GC content at 60%. The common denominator was RNA sample NonT-1 from which the most biased library from every group was prepared. Regarding the rRNA content in libraries, the SENSE kit would perform significantly better than the other two as the percentage of rRNA reads in LX libraries was 1.5% and lower. This could render the combination of ribosomal depletion with Ribocop and library preparation with the SENSE kit ideal for whole transcriptome sequencing. However, it did not necessarily mean that this particular kit would be suitable for the study of lncRNAs and further evidence was needed in order to confirm or disprove this claim.

After evaluating normalized transcript coverage, almost all libraries showed a significantly higher and biased coverage of the 3' end of transcripts. This highlights the importance of high integrity of RNA samples. If the sample quality is poor, polyadenylated transcripts are partially degraded which could result in truncated species with polyA tail still intact. These smaller transcripts might produce cDNA fragments which may, in turn, become overexpressed during the PCR step of the library preparation procedure which could potentially result in deeper coverage of the 3' end of transcripts and introduce the 5' to 3' bias [22]. While all libraries showed normal increase in coverage after 5' end, the remaining portion of the graph hints at different levels of variability in library complexity. NF libraries were characterized with relatively even coverage throughout the genes. This was true more so for NB libraries which showed the most even coverage out of all three groups. This was in sheer contrast with LX libraries which were characterized by irregular coverage resulting in spiky graph curve. However, this was expected since the procedure for this particular approach involves non-random fragmentation which results in lesser variability of fragments generated with the SENSE kit.

Moreover, this was also confirmed upon comparing the aligned reads with Ensembl database which revealed another notable difference between the three methods. The number of hits for each Ensembl biotype of our interest was significantly higher for NB libraries compared to the other two groups, and actually the lowest for LX libraries. This together with the fact that the SENSE kit seemed to prefer coding sequences judging by the significantly higher number of base reads from LX libraries aligning to coding regions would ultimately render the SENSE kit the least suitable for the study of lncRNAs. Another notable difference between these three approaches, which would further affirm this notion, were the percentages of duplicate reads and especially their relation to the expression in reads/kbp. The highest duplication rates were observed in LX libraries with duplicate reads in N1_LX as high as 91.5%. The other two kits performed markedly better which was confirmed by the results both from Picard and dupRadar.

With everything taken into account, the SENSE kit performed well compared to the other two in regard to the normalized GC content and the amount of reads aligning to ribosomal regions but was outperformed by NEBNext in other aspects of our study. Especially important was the discovery that libraries prepared with NEBNext kit had most hits in Ensembl database in every category of our interest including both mRNAs and lncRNAs. This put together with relatively low duplication rates and even transcript coverage made this kit the most applicable for the study of lncRNAs.

To independently validate our results, an expanded cohort of 8 RNA samples with varying levels of integrity was brought to another NGS facility where it underwent ribosomal depletion using RiboZero and library preparation with the NEBNext kit. When comparing resulting VB libraries to NB libraries, the distribution of normalized average GC content was observed to be similar but in case of VB libraries with less pronounced abnormal peaks indicating specific contamination with rRNA. In actuality, this was confirmed using Picard on post-alignment data since the resulting percentages of rRNA reads from VB libraries were lower than those from corresponding libraries G2_NB and N1_NB. The amount of over-represented sequences and that of top over-represented sequence was observed also to be lower for VB libraries compared to NB libraries. After alignment, we noticed slight improvement in mapping statistics for VB libraries, mainly the higher numbers of uniquely mapped reads and reads which overlap only one gene and lower multimapping rates compared to NB libraries. The normalized transcript coverage for VB libraries was similarly even throughout the genes with the exception of 3' end where a much stronger bias resulting in increased depth of sequencing could be observed. On the other hand, duplication rates in VB libraries were still relatively low and comparable to those in NB libraries. All in all, these independent observations confirmed what we had previously seen with NB libraries.

## Conclusions

Looking for new therapeutic targets and clinical biomarkers is vital in case of malignancies with highly unfavorable prognosis such as GBM. Since the discovery of long non-coding transcripts and their multiple regulatory functions, new array of methods including NGS gave rise to a whole new field of genomics and epigentics which focuses on these molecules and their implication in the context of pathophysiological processes. Considering our findings, we conclude that choosing the most suitable approach to library construction for transcriptome sequencing with the aim of studying lncRNAs is highly important in order to retain valuable data on lowly expressed RNA species that could otherwise be permanently lost. Out of the three library preparation kits we tested, NEBNext Ultra II Directional RNA Library Prep for Illumina kit performed best with regard to relatively even transcript coverage, low rates of PCR duplicates and the highest number of hits for biotypes of interest in Ensembl database. We also confirmed the consistency of NEBNext's performance with independent validation on an expanded cohort of patient samples. Therefore, we suggest this approach for lncRNAs sequencing to be used in the studies based on analysis of GBM and brain-tissue specimens.

## Methods

### Patient samples

Samples used in this analysis were part of a larger cohort included in a study focused on sequencing transcriptomes of GBM patients and healthy controls with subsequent bioinformatic and statistical evaluation of the acquired data with the aim of finding putative diagnostic, prognostic or predictive biomarkers and therapeutic targets. Fresh-frozen samples of primary GBM were collected periodically from several Czech neurosurgical departments and independently confirmed by two histopathologists. Non-tumor brain tissues obtained from non-dominant anterior temporal cortexes resected during surgery for intractable pharmacoresistant epilepsy served as healthy controls.

### Isolation and purification of RNA

Tissue samples were mechanically homogenized with ceramic beads and total RNA enriched for small RNA species was then isolated and purified using *mir*Vana miRNA Isolation Kit (Invitrogen) which is based on phenol-chloroform extraction and purification in a filter cartridge. Isolated and purified total RNA was then quantified using both NanoDrop 2000 Spectrophotometer (Thermo Scientific) and diluted to approximately 250 ng/μl for downstream analyses. Diluted RNA was then quantified using Qubit 2.0 Fluorometer in combination with Qubit RNA BR Assay Kit (both Invitrogen) for a more precise measurement.

### RNA quality control

For QC of purified RNA, absorbance ratios A_260_:A_280_ and A_260_:A_230_ were assessed with NanoDrop 2000. The integrity of RNA was evaluated based on RIN acquired via capillary gel electrophoresis performed using Agilent 2200 TapeStation in combination with Agilent RNA ScreenTape System (both Agilent Technologies). The resulting EPG also contained the information about the presence of gDNA carryover from the interphase during RNA isolation which may have a negative impact on downstream applications. In case of such contamination, samples have undergone an additional step of DNase treatment performed with DNA-free DNase Treatment Kit (Invitrogen) before proceeding to the next step.

### Ribosomal depletion and molecular library preparation

For this particular study, 8 RNA samples with varying RIN isolated from 5 GBM and 3 NonT samples were chosen for determining the most suitable library preparation method for NGS. First, all RNA samples underwent rRNA depletion initially done with RiboCop rRNA Depletion Kit V1.2 (Lexogen) and in later stage at the VBCF (www.vbcf.ac.at) with Ribo-Zero rRNA Removal Kit (Human/Mouse/Rat) (Illumina) following the manufacturers' protocols. The input amount of RNA was 500 ng which was in the recommended range for both rRNA removal kits.

In total, three NGS library preparation kits were used to generate molecular libraries from rRNA-depleted RNA samples and compared, namely NEXTflex Rapid Directional qRNA-Seq Kit (Bioo Scientific), SENSE Total RNA-Seq Library Prep Kit (Lexogen) and NEBNext Ultr II Directional RNA Library Prep Kit for Illumina (NEB). All procedures were performed according to the protocols suggested by the manufacturers except VB libraries, which were prepared with NEBNext kit according to the adjusted protocol with minor deviations, which were a result of previous optimization done by the VBCF. Specifically, the time of RNA fragmentation was shortened to 7 minutes for all samples regardless of RIN and the PCR step of library preparation was performed in a real-time set-up with EvaGreen fluorescent dye (Biotium) mixed in to the PCR reaction mix, which, in turn, allowed halting PCR reactions with individual libraries at the proper time in order to avoid overcycling. Samples were multiplexed using suitable molecular barcodes and resulting cDNA pools were processed according to the NextSeq System Denature and Dilute Libraries Guide [23]. The minimum requirements and brief characterization of individual library preparation kits are summarized in Table 6.

**Table 6:** Minimum requirements and brief characterization of three tested library preparation kits. The minimum requirements according to manufacturers' protocols are the quality of pre-depletion RNA and the amount of input rRNA-depleted RNA. The quality of RNA is based either on RNA integrity number (RIN) or the source of RNA, for example formalin-fixed paraffin-embedded (FFPE) tissue blocks. Preceding depletion of rRNA is required for all library prepration procedures.

	NEXTflex (Bioo Scientific)	SENSE (Lexogen)	NEBNext (NEB)
**Minimal quality**	RIN ≥ 5	FFPE	FFPE
**Minimal RNA input**	~1–100 ng rRNA-depleted RNA	~0.6–8.8 ng rRNA-depleted RNA	1–100 ng rRNA-depleted RNA
**rRNA depletion required**	Yes	Yes	Yes
**Fragmentation method**	RNA by divalent cations + heat	Ligation of RT transcript with starter/stopper RT primers	RNA by divalent cations + heat
**cDNA synthesis**	Random primers	Random starter/stopper heterodimers	Random primers
**Strand selection**	Yes	Yes	Yes
**Dual Indexing**	No	Yes	Yes
**Multiplex capacity**	96-plex	384-plex	96-plex
**Strand orientation**	Antisense	Antisense ? Zistím	? Zistím

### Quantification and quality control of molecular libraries

Firstly, the concentration of molecular libraries was measured using Qubit 2.0 Fluorometer in combination with Qubit dsDNA HS Assay Kit (both Invitrogen). Secondly, libraries were analyzed on Agilent 2200 TapeStation in combination with Agilent High Sensitivity D1000 ScreenTape System (both Agilent Technologies) according to the manufacturer's protocol. VB libraries were analyzed on Fragment Analyzer using High Sensitivity NGS Fragment Analysis Kit (both Advanced Analytical). The molarity of individual libraries was calculated using determined concentrations and modal lengths using free online weight-to-moles conversion calculator for nucleic acids [24].

### Next-generation sequencing

Single-read sequencing of CFG libraries with a read length of 75 was performed with NextSeq 500 Sequencing System using NextSeq 500/550 High Output v2 kit (75 cycles) (both Illumina). Paired-end sequencing of VB libraries with a read length of 50 was performed with HiSeq 2500 System using HiSeq SBS Kit V4 kit (50 cycles) (both Illumina). PhiX Control v3 (Illumina) was added at 1% to all pools as an internal control before the sequencing.

### Processing of acquired sequencing data

Subsequent processing of acquired sequencing data was performed with tools in the environment of R-based Bioconductor software. Firstly, the pre-alignment QC of acquired sequencing data contained in FASTQ files was done using FastQC [21]. The sequences were then trimmed for adaptors with the command-line tool cutadapt [25] and all reads were aligned to a reference human genome using STAR [17]. The QC of aligned reads contained in resulting Binary Alignment Map (BAM) files was done with Picard [18]. The same tool was used for determining the percentages of rRNA, mRNA and duplicates. All generated numerical and graphical output was gathered in cohesive reports and exported via MultiQC [26]. Additionally, dupRadar was used for assessment of the distribution of RPK values per gene and relation of duplication rates to expression (RPK) and generating histograms and 2D density scatter plots [20]. Aligned uniquely mapped reads were also compared with Ensembl database (version 93) in order to identify the percentage of distinct molecular biotypes [19].

## Ethics approval and consent to participate

This study was approved by the local Ethical Committee University Hospital Brno (Brno, Czech Republic) and written informed consent was obtained from each patient entering the study.
